# Ketamine and psychotherapy for the treatment of psychiatric disorders: systematic review

**DOI:** 10.1192/bjo.2023.53

**Published:** 2023-05-02

**Authors:** Bess M. Kew, Richard J. Porter, Katie M. Douglas, Paul Glue, Charlotte L. Mentzel, Ben Beaglehole

**Affiliations:** Department of Psychological Medicine, University of Otago, Christchurch, New Zealand; Department of Psychological Medicine, University of Otago, Dunedin, New Zealand

**Keywords:** Ketamine, psychotherapy, systematic review, treatment, psychiatric disorder

## Abstract

**Background:**

Ketamine is an effective short-term treatment for a range of psychiatric disorders. A key question is whether the addition of psychotherapy to ketamine treatment improves outcomes or delays relapse.

**Aim:**

To identify all studies combining psychotherapy with ketamine for the treatment of psychiatric disorders to summarise their effects and make recommendations for future research.

**Method:**

The review protocol was prospectively registered with PROSPERO (registration number CRD42022318120). Potential studies were searched for in MEDLINE, Embase, PsycINFO, SCOPUS, the Cochrane library and Google Scholar. Eligible studies combined ketamine and psychotherapy for the treatment of psychiatric disorders and did not use case reports or qualitative designs. Key findings relating to psychotherapy type, diagnosis, ketamine protocol, sequencing of psychotherapy and study design are reported. Risk of bias was assessed using modified Joanna Briggs critical appraisal tools.

**Results:**

Nineteen studies evaluating 1006 patients were included in the systematic review. A variety of supportive individual and group, manualised and non-manualised psychotherapies were used. The majority of studies evaluated substance use disorders, post-traumatic stress disorder and treatment-resistant depression. Ketamine protocols and sequencing of ketamine/psychotherapy treatment varied substantially between studies. Outcomes were largely positive for the addition of psychotherapy to ketamine treatment.

**Conclusion:**

The combination of psychotherapy and ketamine offers promise for the treatment of psychiatric disorders, but study heterogeneity prevents definitive recommendations for their integration. Larger randomised controlled trials using manualised psychotherapies and standardised ketamine protocols are recommended to clarify the extent to which the addition of psychotherapy to ketamine improves outcomes over ketamine treatment alone.

Ketamine and its derivative S-ketamine improve symptoms of psychiatric disorders including substance use disorders (SUDs), major depressive disorder (MDD) and post-traumatic stress disorder (PTSD) over the short term.^1–3^ A number of barriers limit the translation of these outcome studies to routine care, including: the predominant use of parenteral dosing, significant dissociative side-effects, and the need for repeated dosing with high rates of relapse following treatment cessation.^4–7^ The majority of patients are treated for MDD relapse in the days following a single dose of ketamine.^8^ High relapse rates of depression have also been observed following a course of repeated ketamine doses. For example, Murrough et al^9^ reported a median time to relapse of 18 days following a course of up to six ketamine infusions for treatment-resistant depression (TRD). Similar findings exist for the treatment of PTSD; Albott et al^10^ reported a median time to relapse of 41 days after a course of six ketamine infusions.

The addition of psychotherapy to monoaminergic antidepressants improves outcomes and reduces relapse in MDD^11^ and SUDs,^12^ although the benefits of combination therapy are less clear for PTSD.^13^ It is therefore plausible that the addition of psychotherapy to ketamine could improve outcomes and delay relapse. This is of particular importance given the short-lived positive effects of ketamine treatment. The primary aim of this systematic review was therefore to identify and review studies that combined ketamine and psychotherapy for the treatment of psychiatric disorders. Our goal was to clarify the current evidence for the combined use of ketamine and psychotherapy for psychiatric disorders, highlight areas of importance in this literature and make recommendations for future research.

## Method

This systematic review was guided by the PRISMA checklist^14^ (see Supplementary Appendix A available at https://doi.org/10.1192/bjo.2023.53 for details of the checklist). The study protocol was prospectively registered with PROSPERO (registration number CRD42022318120) and can be accessed at https://www.crd.york.ac.uk/prospero/display_record.php?RecordID = 318120. No ethical approval or informed consent was sought because this was a systematic review of already published studies. The systematic review was conducted according to the prospectively registered protocol with the exception of quality assessment. The protocol proposed using the Cochrane risk of bias (RoB) 2^15^ and ROBINS-I^16^ tools. After trialling these tools in a sample of the included papers, we chose to complete RoB assessment using the Joanna Briggs critical appraisal tools.^17^ This decision was made because the RoB 2 and ROBINS-I tools were not well-suited to the range of study types identified by our review (including randomised control trials (RCTs), quasi-experimental studies, single-arm studies and case series). We considered other commonly used RoB tools but chose to use the Joanna Briggs suite of tools for consistency rather than apply multiple tools from different origins. Although this decision was made after included studies had been identified by the screening process, it occurred prior to detailed review of the studies. Supplementary Appendix B provides details of the RoB assessments. The study protocol included the option of meta-analysis. However, the highly heterogeneous nature of identified studies meant we chose not to do so.

### Inclusion criteria

Studies were eligible for inclusion if they reported the use of ketamine or S-ketamine in conjunction with psychotherapy for the treatment of a psychiatric disorder. Psychotherapy was broadly defined and included any psychological treatment that uses regular communication between a patient and a psychologist, counsellor or other trained mental health professional for the purpose of improving well-being related to a psychiatric disorder. Psychiatric disorder was defined as any psychiatric disorder diagnosed using DSM or ICD diagnostic criteria. Clinical diagnoses made by expert clinicians outside of these classifications were also included. Eligible study designs included RCTs, cohort studies, and case series, open-label and feasibility studies. Single case reports and qualitative studies were not included in the review. Studies were required to compare measures of psychiatric disorder using validated rating scales pre- and post-ketamine and psychotherapy treatment.

### Search and screening strategy

The search strategy combined a search for ketamine and its derivates and a search for psychotherapy using the Boolean classifier AND. The search was undertaken with assistance from a research librarian and was limited to English language and human trials. Supplementary Appendix C reports the search strategies used for each database. Potential studies were searched for in MEDLINE, Embase, PsycINFO, SCOPUS, the Cochrane library and Google Scholar. The study period was from database conception to May 2022. In addition, the reference lists of eligible papers were searched, and a forward citation search was undertaken.

Authors B.B. and B.M.K. independently screened titles and abstracts in accordance with the inclusion criteria using Covidence, the Cochrane Foundation's online platform for systematic review management.^18^ B.B. and B.M.K. also independently reviewed full-text articles for potential inclusion, and disagreements were resolved by discussion between the two authors at each step. B.M.K. conducted the data extraction using a data extraction sheet that was reviewed and adjusted after a trial period. B.B. independently completed data extraction, and any disagreements were resolved with B.M.K. through discussion.

### Analysis

Substantial between-study heterogeneity prevented meta-analysis or the use of standardised metrics to report outcomes for the included studies. We therefore summarise findings according to categories that highlight key elements of the included studies to better allow consideration. The categories were as follows.

(a) Psychotherapy: the range of psychotherapies used in the included studies are described including any specific elements unique to ketamine treatment.

(b) Diagnosis: outcomes of included studies are reported according to diagnostic groupings. Results from RCTs then studies with larger sample sizes are reported first. Where possible, key statistics that highlight the magnitude of change pre–post treatment or between ketamine and ketamine psychotherapy arms are provided.

(c) Ketamine protocol: ketamine protocols for the included studies are described.

(d) Scheduling of ketamine and psychotherapy treatments: sequencing strategies for the use of ketamine and psychotherapy are reported.

(e) Study design and timing of outcome measurement: choice of comparators and timing of outcome measurement are reported to assist focus on whether psychotherapy improves outcomes in addition to ketamine treatment and inform the duration of any positive effects.

(f) Risk of bias: the RoB of included studies was assessed using the Joanna Briggs critical appraisal tools.^17^ This included using an adapted form for the single-arm studies in the review based on collating relevant items from other study types. The adaptation was undertaken to ensure the single-arm studies could be similarly assessed for bias in study inclusion, patient characteristics, intervention, outcome measurement, analysis and potential confounders. Three RoB categories (low, moderate and high) were created based on the distribution of positively endorsed items in the appropriate critical appraisal checklist for the study type. These categories were created prior to scoring of the RoB tools, to assist the reader. Studies with ≤30% of endorsed items were rated high RoB, studies with >30% to ≤70% endorsed items were rated moderate RoB and studies with >70% were rated low RoB. This approach is similar to that of other authors who regarded a score of 70% and ≥7 positively endorsed items on Joanna Briggs Institute scales as an indicator of quality.^19,20^ Authors B.B. and B.M.K. independently completed the RoB assessments for each study, and a consensus rating for each study was reached through discussion.

## Results

**Fig. 1:**
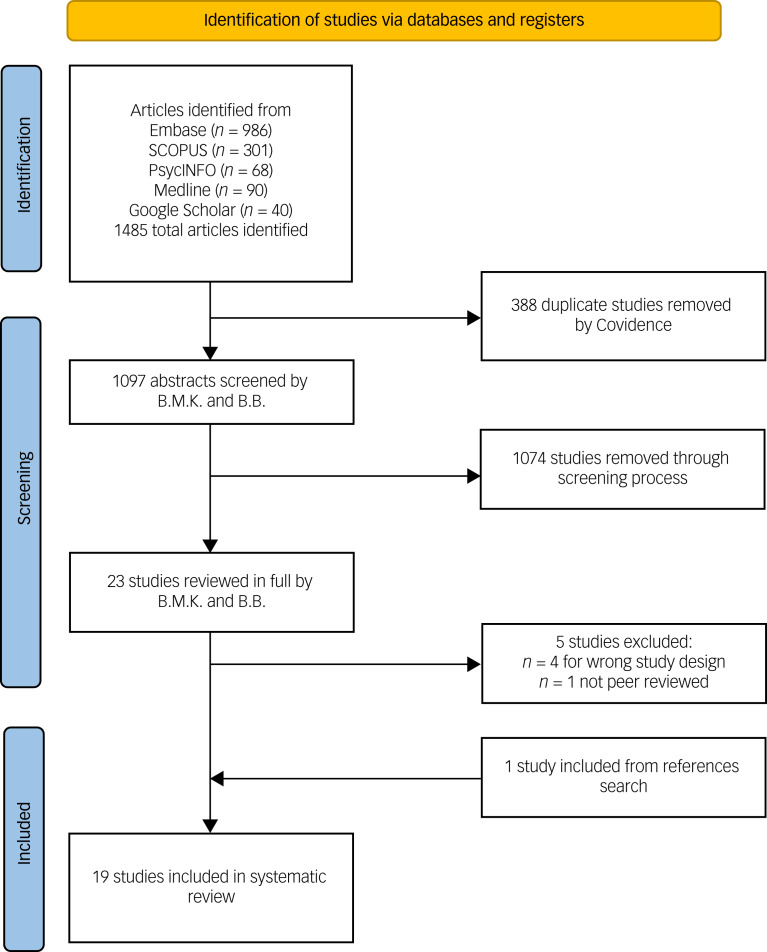
PRISMA flow diagram for the search process.

Figure 1 is the PRISMA flow diagram for the screening and inclusion process. Database searches were undertaken between 28 April 2022 and 2 May 2022. A total of 1485 abstracts were screened after removing duplicates, resulting in 23 papers for full-text review. After full-text screening, 19 studies were included in the systematic review. These studies comprised ten single-arm studies, eight RCTs and one non-RCT study. A total of 1008 patients were represented in the systematic review. The RCTs evaluated 337 patients receiving ketamine with psychotherapy compared with 252 control participants. A further 419 patients received ketamine and psychotherapy in the non-randomised studies. The sample sizes for included studies ranged from five to 235 participants. Tables 1 and 2 provide details of the included studies including diagnostic grouping, ketamine and psychotherapy treatments, key results and the outcome of the RoB assessments.

**Table 1:** Controlled trials

Study	Diagnostic group	Sample size	Ketamine protocol	Psychotherapy	Treatment/comparator	Type of blinding	Outcome measure(s)	Key results	Risk of bias
Dakwar et al, 2019	CD	*N* = 55	0.50 mg/kg ketamine infusion in once in W1	MBRP received daily for 4 days in W1, biweekly for 4 weeks	Ketamine + MBRP/ midazolam + MBRP	Double blind	Urine toxicology	Significant difference between proportion of abstinent participants in ketamine group (48.2%) versus midazolam group (10.7%) in last 2 weeks of treatment. After controlling for route of use, odds of EOT abstinence in ketamine group was 5.7 times that of midazolam group (95% CI 1.3–25.1)	Low
Dakwar et al, 2020	AUD	*N* = 40	0.71 mg/kg ketamine infusion, once in W2	MET once weekly for 5 weeks, additional session in W2	ketamine + MET/ midazolam + MET	Double blind	TLFB	Significant difference between proportion of abstinent participants in ketamine group compared with midazolam group at 21 days after ketamine infusion	Low
Grabski et al, 2022	AUD	*N* = 96	0.80 mg/kg ketamine infusion, three times over 3–9 weeks	MBRP six times over 3–9 weeks	Ketamine + MBRP / ketamine + AE/saline + MBRP/saline + AE	Double blind	TLFB, rate of relapse 6 months follow-up	Significant difference between proportion of alcohol-abstinent days at 6 months in the ketamine group compared with the placebo group, pooled across therapy conditions (mean difference = 10.10, 95% CI 1.1–19.0. No difference in relapse rates between groups	Low
Krupitsky & Grinenko, 1996 (non- randomised)	Alcoholism	*N* = 211	2–3 mg/kg i.m. ketamine (in addition to i.m. aethimizol and bemegride, and oral nimodipine)	Psychotherapy leading up to and during ketamine session. Group therapy the day after ketamine session	Ketamine + psychotherapy/TAU	No blinding	Rate of abstinence at 1 year follow-up	Abstinence at 1 year follow-up was observed in 65.8% of participants in the ketamine group, compared with 24.0% in the TAU group	Moderate
Krupitsky et al, 2002	HD	*N* = 70	2.0 mg/kg i.m. ketamine, administered once	Psychotherapy 10 h before ketamine, 1.5–2 h during ketamine session, 5 h after ketamine	Ketamine + psychotherapy/low-dose ketamine (0.2 mg/kg i.m.) + psychotherapy	Double blind	Rate of abstinence at 2 year follow-up	Abstinence at 2 year follow-up was significantly greater in the ketamine group (~17.5%) compared with the comparison group (~2.5%). Exact proportions not reported	Low
Krupitsky et al, 2007	HD	*N* = 59	2.0 mg/kg i.m. ketamine. Frequency differed between groups	Psychotherapy at the same time as ketamine, in addition to addiction counselling. Frequency differed between groups	Three ketamine + psychotherapy sessions/1 ketamine + psychotherapy session	Evaluator blind	Rate of abstinence at 1 year follow up	Significant difference in proportion of abstinent participants in multiple (50%) versus single (22.2%) ketamine + psychotherapy sessions at the 1 year follow-up	Low
Pradhan et al, 2017	PTSD	*N* = 10	0.50 mg/kg ketamine infusion once in W1	TIMBER three times in W1, weekly in W2–W10	Ketamine + TIMBER/ saline + TIMBER	Double blind	CAPS, PCL	No significant difference between ketamine + TIMBER and saline + TIMBER groups in regard to percentage change scores in CAPS or PCL	Moderate
Pradhan et al, 2018	PTSD	*N* = 20	0.50 mg/kg ketamine infusion once in W1	TIMBER three times in W1, weekly in W2–W10	Ketamine + TIMBER/saline + TIMBER	Double blind	CAPS, PCL	At 24 h post infusion, all participants experienced a significant remission of symptoms (>60% reduction) as measured by both CAPS and PCL scores. No significant difference in response between the two groups at 24 h. However, there was a significant difference between number of days that the effect was sustained in the ketamine group (*M* = 34.44, s.d. = 19.12) compared with the comparator group (*M* = 16.50, s.d. = 11.39).	Low
Wilkinson et al, 2021	TRD	*N* = 28	0.50 mg/kg ketamine infusions, six infusions within W1–W3	CBT twice weekly in W4 and W5, once weekly W6–W17	Ketamine + CBT/ketamine + TAU	Administrator blind	MADRS	Only responders were randomised into either CBT or TAU. No significant time by treatment interaction when measured with MADRS (*d* = 0.65, 95% CI −0.55 to 1.82). However, a significant time by treatment interaction was observed when measured by QIDS, with CBT showing greater sustained improvements in symptoms compared with TAU (*d* = 0.71, 95% Cl = −0.30, 1.70	Moderate

AUD, alcohol use disorder; CD, cocaine dependence; HD, heroin dependence; PTSD, post-traumatic stress disorder; TRD, treatment-resistant depression; AE, alcohol education; CBT, cognitive–behavioural therapy; i.m., intramuscular; KPT, ketamine psychedelic therapy; MET, motivational enhancement therapy; MBRP, mindfulness-based relapse prevention; TAU, treatment as usual; TIMBER, trauma interventions using mindfulness-based extinction and reconsolidation; W, week; CAPS, Clinician Administered Scale for DSM; EOT, end of treatment; MADRS, Montgomery–Åsberg Depression Rating Scale; PCL, PTSD Checklist; QIDS, Quick Inventory of Depressive Symptomatology; TLFB, timeline follow back.

**Table 2:** Single-arm trials

Study	Diagnostic group	Sample size	Ketamine protocol	Psychotherapy	Outcome measure/s	Type of blinding	Key results	Risk of bias
Azhari et al, 2021	CUD	*N* = 8	0.71 mg/kg ketamine infusion at W2, and 1.41 mg/kg ketamine infusion at W4 for those struggling to maintain abstinence (*n* = 3)	MET at W1, day before ketamine and afternoon of ketamine. MET once the day before second ketamine infusion, if applicable. MBRP twice weekly in W3–W6	TLFB	Single blind	Significant decrease in mean number of days of cannabis use in last 7 days from baseline (*M* = 5.12, s.d. = 1.89) to the week following first infusion (W3, *M* = 0.75, s.d. = 1.17), and to EOT (W6, *M* = 0.50, s.d. = 0.93).	Moderate
Dames et al, 2022	Mixed	*N* = 94	1–1.5 mg/kg i.m. ketamine at W4, W5 and W7 in group setting	CoP (group) once weekly for 12 weeks	PHQ-9, PCL-5, GAD-7, B-IPF	No blinding	In participants who screened positive for the target diagnoses, 91% had improvements in GAD, 76% improved in depression, 86% of those with PTSD at baseline no longer met diagnostic criteria and 92% had improvements in life/work functionality	High
Davis et al, 2021	PTSD	*N* = 18	100–200 mg sublingual ketamine administered before each therapy session	BCT once weekly for 6 weeks	PC-PTSD-5, PHQ-2, GAD-2, SDS	No blinding	Significant decrease in mean PHQ-2 scores from intake (*M* = 3.88, s.d. = 2.30) to EOT (*M* = 2.72, s.d. = 2.44, *d* = −0.53). Improvements in PC-PTSD (*d* = −0.48) and SDS (*d* = −0.52) failed to reach significance. No change in GAD-2	Moderate
Dore et al, 2019	Mixed	*N* = 235	i.m. and/or sublingual ketamine between 1 and 25 times, doses individualised	Non-manualised psychotherapy	BDI, HAM-A	No blinding	Significant decrease in mean BDI scored from baseline (*M* = 26.55) to EOT (*M* = 15.31). Significant decrease in mean HAM-A scores from baseline (*M* = 20.35) to EOT (*M* = 15.03)	High
Keizer et al, 2020	PTSD	*N* = 11	2 μg/kg/min ketamine infusion initially, dose increased 1–2 μg/kg/min every 3–4 h until 11–15 μg/kg/min achieved. Infusion ran 96 h on average	Exposure-based PTSD therapy daily for 5 days	PCL-5	No blinding	Significant decrease in mean PCL scores from pre-treatment (*M* = 55.33, s.d. = 11.52) to post treatment (*M* = 27.11, s.d. = 12.36, 95% CI 13.11–43.33), with a large effect size (*d* = 1.44)	High
Robison et al, 2022	ED	*N* = 5	25 mg i.m. ketamine once in W1, doses individualised thereafter at each session held once weekly in W2–W4	Group and individual therapy as part of routine in-patient ED treatment	PHQ-9, GAD-7	No blinding	From baseline to EOT, *n* = 4 participants experienced clinically significant improvements in PHQ-9 scores, *n* = 2 participants experienced clinically significant improvements in GAD-7 scores	Moderate
Rodriguez et al, 2016	OCD	*N* = 10	0.50 mg/kg ketamine infusion, once in W1	Exposure-based CBT, ten sessions over 2 weeks	YBOCS	Single blind	Significant decrease in mean YBOCS scores from baseline (*s* = 28.80, s.d. = 4.60) to W2 (*M*= 18.30, s.d. = 8.90), and from baseline to 2 weeks after EOT (*M*= 21.90, s.d. = 9.80)	Moderate
Shiroma et al, 2020	PTSD	*N* = 10	0.50 mg/kg ketamine infusion weekly for 3 weeks	PE once weekly 24 h after infusion, once weekly for up to 7 weeks thereafter	CAPS-5	No blinding	Significant decrease in CAPS-5 scores from baseline to EOT (*t* 11 = 4.21, *P* = .001, −15.25, 95% CI 7.27–23.23, *d* = 1.21)	Low
Wilkinson et al, 2017	TRD	*N* = 16	0.50 mg/kg ketamine infusion twice weekly for 2 weeks	CBT twice weekly for 2 weeks, once weekly for an additional 8 weeks	MADRS	No blinding	50% of the total sample achieved response (≥50% in MADRS), and 43.8% achieved remission (MADRS score ≤9)	Low
Zydb & Hart, 2021	TRD	*N* = 10	75 mg oral ketamine, once in W1 and W5	TRIP psychotherapy 20 min after ketamine dose, once in W1 and in W5	PHQ-9	No blinding	Significant decrease in PHQ-9 from baseline (*M* = 17.90, s.d. = 5.10) to EOT (W5, *M* = 9.50, s.d. = 6.60)	Moderate

CUD, cannabis use disorder; GAD, generalised anxiety disorder; OCD, obsessive–compulsive disorder; PTSD, post-traumatic stress disorder; TRD, treatment-resistant depression; BCT, body-centred therapy; CBT, cognitive–behavioural therapy; CoP, community of practice; i.m., intramuscular; KAP, ketamine-assisted psychotherapy; KEP, ketamine-enhanced psychotherapy; MBRP, mindfulness-based relapse prevention; MET, motivational enhancement therapy; PE, prolonged exposure therapy; SDS, Sheehan Disability Scale; TRIP, therapeutic reset of internal processes; W, week; BDI, Beck Depression Inventory; B-IPF, Brief Inventory of Psychosocial Functioning; PC-PTSD-5, Primary Care PTSD Screen for DSM-5; CAPS-5, Clinician-Administered PTSD Scale for DSM-5; EOT, end of treatment; GAD-2, Generalised Anxiety Disorder 2; HAM-A, Hamilton Rating Scale for Anxiety; MADRS, Montgomery–Åsberg Depression Rating Scale; PCL-5, PTSD Checklist for DSM-5; PHQ-2, Patient Health Questionnaire, TLFB, timeline follow back; YBOCS, Yale–Brown Obsessive Compulsive Scale; ED, eating disorder.

### Psychotherapy type

#### Mindfulness-based relapse prevention

Three of the 19 studies evaluated mindfulness-based relapse prevention (MBRP) and ketamine.^21–23^ MBRP is a manualised treatment that combines components of cognitive–behavioural therapy (CBT), stress reduction and lessons to develop mindfulness skills.^24^ MBRP aims to teach patients to recognise and address early warning signs of substance relapse.^25^

#### Cognitive–behavioural therapy

Three studies evaluated CBT and ketamine.^26–28^ CBT combines psychoeducation, cognitive restructuring and behavioural activation to address symptoms of psychiatric disorders.^29^

#### Ketamine psychedelic therapy

Three studies evaluated ketamine psychedelic therapy (KPT).^30–32^ KPT is a three-stage ketamine and psychotherapy protocol developed to treat SUDs.^33^ Stage 1 involves preliminary individual psychotherapy to prepare the patient for the ketamine treatment and explores factors relating to their substance misuse history. Stage 2 consists of ketamine dosing and psychotherapy while the patient is under the influence of ketamine. The psychotherapy in this stage revisits themes explored in stage 1 to integrate factors identified earlier and harness the potential for change. The final stage starts the day after the ketamine session and involves discussion, interpretation, and integration of the patient's experiences in stage 2.

#### Motivational enhancement therapy

Two studies evaluated motivational enhancement therapy (MET) and ketamine.^21,34^ MET, also known as motivational interviewing, is a counselling technique used to enhance intrinsic motivation by addressing ambivalence towards behaviour change.^35^

#### Trauma interventions using mindfulness-based extinction and reconsolidation

Two RCTs evaluated trauma interventions using mindfulness-based extinction and reconsolidation (TIMBER) and ketamine.^36,37^ TIMBER therapy incorporates components of CBT, yoga and mindfulness-based graded exposure to treat PTSD.

The remaining psychotherapies were only used in single studies. These were a mix of manualised and non-manualised therapies (see Tables 1 and 2 for details of the therapy type).

### Diagnosis

#### Substance use disorders

Seven of the 19 studies evaluated treatment of SUDs. Three studies were for the treatment of alcohol use disorder (AUD), alcohol dependence or alcoholism.^23,32,34^

Dakwar et al^34^ completed an RCT comparing MET and ketamine with MET and midazolam for patients with AUD (*n* = 40). Patients received six MET sessions within 5 weeks. At 12 days post-ketamine infusion, there was a significantly greater proportion of alcohol-abstinent patients in the ketamine group (52.9%) compared with the midazolam group (40.9%).

Grabski et al^23^ completed a four-arm RCT (*n* = 96) for AUD comparing combinations of ketamine or saline treatment with MBRP or alcohol education (an active therapy control). Patients allocated to receive MBRP received six sessions over 3–6 weeks. At 6 months after the end of treatment, higher rates of abstinence were reported for ketamine groups compared with saline groups when the results were pooled across therapy conditions (mean difference = 10.1, 95% CI 1.1–19.0). However, no significant difference in alcohol-abstinent days was present between the ketamine plus MBRP group and the ketamine plus alcohol education group.

Krupitsky and Grinenko^32^ evaluated KPT for the treatment of alcoholism (*n* = 211). Patients received the KPT protocol at the end of a 3-month in-patient treatment plan that involved treatment of alcohol withdrawal syndrome and comorbid psychiatric and/or somatic disorders with individual and group psychotherapy. In this study, stage 3 of KPT was undertaken in groups with patients who had received ketamine the previous day. Outcomes were compared between those who had received KPT and a comparison group of in-patients that had not received KPT. At 1 year post-treatment, abstinence was reported in 65.8% of patients in the KPT group compared with 24.0% in the comparison group.

Two RCTs evaluated heroin dependence.^30,31^ In the study of Krupitsky et al,^31^ all patients (*n* = 70) received KPT, although stage 3 was provided individually. Patients were randomly assigned to receive high-dose ketamine or low-dose ketamine during stage 2 of KPT. The rate of heroin abstinence at 2 years post-intervention was approximately 17.5% in the high-dose ketamine group and approximately 2.5% in the low-dose ketamine group.

In a separate RCT, Krupitsky et al^30^ treated heroin-dependent patients (*n* = 59) with KPT, with the third stage provided individually. Patients were then randomly assigned to receive two further addiction counselling sessions at monthly intervals or two additional stage 2 ketamine doses and addiction counselling at monthly intervals. Patients in the multiple KPT group also received an additional two psychotherapy sessions after each ketamine dosing to integrate their experiences. At 1-year follow-up, 50.0% heroin abstinence rates were reported in the multiple session group compared with 22.2% in the single session group.

One RCT evaluated cocaine dependence.^22^ In this study, patients (*n* = 55) received 12 MBRP sessions over 5 weeks. At the end of treatment, abstinence from cocaine was observed in 48.2% of patients receiving ketamine and MBRP compared with 10.7% of patients receiving midazolam and MBRP. After controlling for route of use, the odds of abstinence in the ketamine group were 5.7 times those of the midazolam group (95% CI 1.3–25.1). One single-arm study evaluated cannabis use disorder.^21^ Patients (*n* = 8) received ketamine treatment combined with two sessions of MET, followed by six sessions of MBRP. At 12 days post-infusion, there was a significantly greater proportion of cannabis-abstinent patients in the ketamine group compared with the midazolam group.

#### Post-traumatic stress disorder

Five studies evaluated patients with PTSD, one of which also included patients with generalised anxiety disorder and MDD.^38^ Two very small RCTs (*n* = 10–20)^36,37^ evaluated TIMBER therapy and ketamine for the treatment of PTSD. Neither study reported a significant difference in PTSD symptoms between groups at 24 h after treatment.^36,37^ However, Pradhan et al^37^ reported a longer treatment effect in the ketamine group (*M* = 34.44, s.d. = 19.12) compared with the comparator group *(M* = 16.50, s.d. = 11.39) following the intervention.

Two small (*n* = 10–11) open-label studies evaluated ketamine and psychotherapy for PTSD.^39,40^ Both studies reported large effect size improvements in PTSD scores (*d* = 1.44, and *d* = 1.21) from pre-post treatment.^37,38^ A retrospective analysis of clinical records of patients receiving body-centred therapy for PTSD reported moderate effect-size improvements in PTSD scores (*d* = −0.48) that failed to reach statistical significance.^36^

#### Treatment-resistant depression

Three studies evaluated TRD.^27,28,41^. Wilkinson et al^28^ conducted an open-label study of TRD patients (*n* = 16) involving 12 sessions of CBT over a 10-week period following ketamine treatment. Fifty per cent of the study population responded to the intervention, and 43.8% achieved remission. Wilkinson et al^27^ then completed a follow-up RCT for patients with TRD (*n* = 28). Patients who responded to an initial ketamine dose were allocated to receive 15 sessions of CBT over 17 weeks or treatment as usual (TAU). TAU consisted of regular meetings with a study physician for concomitant medication management. Wilkinson et al^27^ did not report significant between-group differences favouring the addition of psychotherapy to ketamine when measured by a clinician-rated depression scale (Montgomery–Åsberg Depression Rating Scale, MADRS), although the effect size was moderate (*d* = 0.65, 95% CI −0.55 to 1.82). However, a significant difference in favour of ketamine and psychotherapy treatment was observed when the Quick Inventory of Depression Symptomatology self-report depression scale was used (*d* = 0.71, 95% CI −0.30 to 1.70).

The remaining TRD study was an open-label study (*n* = 10) that reported significant improvements in self-reported depression symptoms at the end of ketamine and psychotherapy treatment.^41^

#### Other diagnostic groups

The remaining four studies were single-arm studies that evaluated patients with eating disorders and comorbid generalised anxiety and depression symptoms,^42^ obsessive–compulsive disorder (OCD)^26^ and mixed diagnostic samples.^43,44^

Dore et al^44^ retrospectively analysed data from patients (*n* = 235) who had received ketamine and psychotherapy across three private psychiatric practices. Patients in this study had varying diagnoses including MDD, PTSD, complex PTSD, attention-deficit hyperactivity disorder, anxiety disorders and SUDs. The number of in-office ketamine and psychotherapy sessions ranged from 1–25. Some patients were also prescribed sublingual ketamine to take at home between in-office ketamine and psychotherapy sessions. Ketamine dose, route and frequency and psychotherapy frequency were individualised depending on the patients’ diagnosis and circumstances. Symptom rating scales were administered at intake and again at discharge from treatment. Significant decreases in the Beck Depression Inventory (*M* = 26.55 at baseline, *M* = 15.31 post treatment, *P* < 0.0001) and the Hamilton Rating Scale for Anxiety (*M* = 20.35 at baseline, *M* = 15.03 post treatment, *P* < 0.0001) were both reported at the end of treatment.

Dames et al^43^ administered ketamine in a group setting at weekly intervals for 3 weeks to patients (*n* = 94) with treatment-resistant psychiatric disorders. Processing took place within the group after the effects of the drug had subsided (approximately 90 min after dosing). Processing included conversation about the patients’ respective ketamine experiences. This was in addition to psychotherapy leading up to and following the ketamine sessions. By treatment end, improvements were reported in 91% of patients with generalised anxiety disorder symptoms, and 76% of patients with depression symptoms. Eighty-six per cent of patients with a baseline PTSD diagnosis were reported to no longer meet diagnostic criteria.

One small (*n* = 10) open-label study examined the treatment of OCD.^26^ This study provided a single intravenous (i.v.) ketamine infusion followed by exposure-based CBT. The mean estimated Yale–Brown Obsessive Compulsive Scale score was significantly lower at week 2 (difference = −10.75 points, s.e. = 1.44, *P* < 0.0001) and at week 4 (difference = −6.88, s.e. = 2.61, *P* = 0.01). A wide variation in response among individuals was reported in this study. An eating disorders study (*n* = 5)^42^ evaluated a course of ketamine alongside routine group and individual therapy. This study reported significant improvements in depression scores for four participants and significant improvements in anxiety scores for two participants following treatment with ketamine and psychotherapy.

### Ketamine protocol

#### Intravenous

Eleven of the 19 studies examined psychotherapy and ketamine administered via i.v. infusion.^21–23,26–28,34,36,37,39,40^ Doses ranged from 0.50 mg/kg to 1.41 mg/kg in the case of non-response. Treatment regimes include single and repeated infusions in combination with therapy.

#### Intramuscular

Six studies evaluated psychotherapy plus intramuscular (i.m.) ketamine injections.^30–32,42–44^. Treatment doses started at 25 mg in the ED population^42^ and were typically in the 1–2 mg/kg range. Dosing frequency ranged from single ketamine dose protocols^32^ to the case series reported by Dore et al^44^ with up to 25 ketamine treatment sessions.

#### Oral/sublingual

Two single-arm studies evaluated psychotherapy and sublingual ketamine^38^ or oral ketamine.^41^ Zydb and Hart^41^ examined oral doses of 75 mg administered once a week for 6 weeks. Davis et al^38^ examined sublingual doses between 100 and 200 mg administered three times over 6 weeks.

### Scheduling of psychotherapy and ketamine within the study design

Ketamine and psychotherapy were delivered in various sequences in the included studies. Nine studies involved the delivery of psychotherapy at the time of ketamine dosing (when participants were acutely affected by ketamine.^30–32,38,39,41–44^

One study (Grabski et al^23^) delivered ketamine and psychotherapy concurrently, whereby therapy sessions were timed so that they preceded ketamine administration in addition to occurring approximately 24 h later.

Nine studies delivered an initial package of ketamine treatment (sometimes combined with psychotherapy) followed by further psychotherapy.^21,22,26–28,34,36,37,40^

### Study design and timing of outcome measurement

The RCT by Wilkinson et al compared ketamine treatment plus CBT with ketamine treatment with TAU.^27^ As a consequence, this study was ideally situated to report on the additional benefits of providing psychotherapy with ketamine treatment. The comparison groups for RCTs by Dakwar et al,^22,34^ Grabski et al,^23^ Pradhan et al^36^ and Krupitsky et al^30,31^ included active medication controls, saline controls, high/low doses of ketamine and single/repeated ketamine doses. As a consequence, these studies were better suited to distinguishing between medication effects and the benefits of additional psychotherapy.

The single-arm studies evaluated packages of ketamine and psychotherapy, but, owing to the absence of control arms, these studies evaluated the combined treatments as opposed to the individual elements.^21,26,28,38–44^

Most studies completed outcome measurements at the end of the treatment package or shortly afterwards. The studies by Grabski et al^23^ and Krupitsky et al^30–32^ reported outcomes at time points distant from the end of psychotherapy and ketamine treatment and were therefore able to report relapse in patients with SUDs. They followed patients for 18 months after ketamine treatment and were therefore able to report maintenance treatment effects.

### Risk of bias

Supplementary Appendix B reports the details of the RoB assessments. Seven of the 19 studies were rated as low RoB; six of these were controlled trials and two were single-arm studies.^22,23,28,30,31,34,37,40^ Low RoB was characterised by adequate randomisation and blinding procedures (where applicable), sufficient detail in reporting study design, standardised disorder measurement and appropriate statistical analyses.

Nine studies were rated as moderate RoB, of which three were controlled trials and five were single-arm studies.^21,26,27,32,36,38,41,42^ Moderate RoB indicated areas of potential weakness in study design, method, data collection or statistical analysis.

Three remaining studies were rated as high RoB, and all were single-arm studies.^39,43,44^ High RoB was attributed to these studies owing to a lack of detail on inclusion and exclusion criteria, baseline characteristics and the intervention protocol. In the studies of Dames et al^43^ and Dore et al,^44^ treatment protocols differed between patients.

## Discussion

We identified 19 studies treating 1008 patients with ketamine and psychotherapy across nine psychiatric disorders. The majority of the studies reported positive outcomes associated with ketamine and psychotherapy treatment. However, heterogeneity in diagnoses, psychotherapies and ketamine protocols meant that robust conclusions for clinical recommendations could not be made.

The largest studies evaluated the treatment of SUDs. For AUD, ketamine treatment performed better than a saline control (Grabski et al 2022^23^), and KPT performed better than usual treatment (Krupitsky and Grinenko^32^). For heroin dependence, patients allocated to higher-dose ketamine and repeated ketamine/psychotherapy groups had better outcomes than those in comparator groups.^30,31^

The diagnoses identified by this review were AUD/alcohol dependence/alcoholism, heroin dependence, cocaine dependence, cannabis use disorder, PTSD, TRD, eating disorders and OCD. There may be shared features across these disorders, such as the trait of neuroticism,^45^ which are targeted by ketamine and psychotherapy.^46^ It is also possible that non-specific treatment expectation effects mediate the benefits reported and that the specific benefits of combining ketamine and psychotherapy are at risk of being overestimated. Supporting this possibility is the finding that positive early-stage intervention studies are more likely to be reported and that publication bias exists in the early stages of researching new clinical interventions.^47^ We note that ketamine is likely to be associated with significant expectation biases,^48^ and the addition of psychotherapy may amplify the already intense experience and possible placebo effect of ketamine.

The review reported a total of 12 psychotherapies. These included manualised and non-manualised psychotherapies, delivered individually or in group settings. This heterogeneity meant that it was unclear whether there is a preferred therapy protocol for combination with ketamine treatment. The manualised psychotherapies (MBRP, MET, CBT, TIMBER) are the most straightforward to replicate and better suited to research protocols. We therefore recommend their use, as well as the use of existing therapies with established indications for target disorders over unstructured psychotherapies and *de novo* therapies, in order to grow a more consistent research base in this area. The use of manualised therapies will also strengthen the translation of studies into routine clinical care by service providers.

A number of studies were not designed to delineate the individual effects of psychotherapy and ketamine. These included the single-arm studies evaluating the treatment combination and the RCTs that were controlled using placebos as opposed to a therapy control. Only the study by Wilkinson et al compared ketamine plus CBT with ketamine plus TAU to demonstrate the benefits of adding therapy to ketamine treatment. In order to further advance this area of research, we suggest that ketamine and psychotherapy studies consider the most appropriate comparator arms. A recent study published by Price et al^49^ is worth highlighting for design purposes. This study (*n* = 154) randomised patients with TRD to ketamine plus automated self-association training (ASAT), ketamine plus sham ASAT or saline plus ASAT. Although ASAT did not meet our therapy definition, the design of this study meant the separate effects of ketamine and ASAT could be distinguished. In addition, ASAT may point to a way forwards for the wider delivery of therapeutic interventions.

There was an extensive range of ketamine protocols applied by the studies. These included single-dose treatments, oral/i.m./i.v. administration and repeated dosing. The variation identified meant that it was difficult to conclude whether there is a preferred ketamine protocol for combination with psychotherapy. We therefore recommend that future researchers use standardised ketamine protocols that have been shown to be efficacious in the short-term treatment of psychiatric disorders. This will enable the additional benefits of psychotherapy to be delineated and will be easier to replicate. Although the evidence base for oral ketamine is at an earlier stage than that for parenteral ketamine,^50^ we suspect that the advantages of oral ketamine are significant when ketamine treatments are considered for more widespread use, and this factor should also be considered.

A range of psychotherapy sequencing protocols were also identified, with psychotherapy preceding, following and being provided at the same time as ketamine treatments. A number of rationales exist for the variety of sequencing. These include preparation of the patient for ketamine treatment, direct influence of psychotherapy on the patient's ketamine experience and taking advantage of opportunities to change following ketamine response. It is still unclear whether the dissociative experience of ketamine augments or hinders the psychotherapy component. At this stage, there is not sufficient evidence to support a particular sequencing protocol for future ketamine and psychotherapy studies, but this area is important to resolve. A systematic review by Joneborg et al^51^ discussed possible mechanisms for the efficacy of combined use of ketamine and psychotherapy, although only a small number of studies were identified that had reported on this topic. This review also noted that despite possible synergistic effects, evidence for this possibility remained speculative.^51^ We therefore recommend that future research considers comparing different sequencing protocols and includes design elements that address mechanisms of effect.

The timing of outcome measurement varied substantially between studies. Given the established short-term efficacy of ketamine treatment, a key question is whether psychotherapy delays relapse or improves outcomes over the medium to longer-term. The studies by Grabski et al (2022)^23^ and Krupitsky et al (1996, 2002, 2007)^30–32^ suggest that the addition of psychotherapy to ketamine treatment improves long-term abstinence rates for the treatment of AUD and heroin dependence. Pradhan et al (2018)^37^ reported longer term outcomes but did not include a therapy control arm. The other 14 studies did not include sufficient follow-up periods to clarify whether psychotherapy improves outcomes over the medium to longer term. We therefore recommend that future research in this area includes outcome measurement at time points distant to treatment end for medium- to long-term benefits to be clarified.

### Limitations

This systematic review included all studies that evaluated ketamine treatment in combination with any psychotherapy for the treatment of any psychiatric disorder. It therefore provides a high-level overview of the extant literature. We believe this approach suited the current status of the field and recognise that comparisons between specific therapies for differing indications are not yet able to be made. Like other researchers, we were challenged by the lack of a gold-standard RoB assessment tool for quantitative studies.^52,53^ We elected to use the Joanna Briggs critical appraisal tools (modified for the single-arm studies) for the RoB assessments as these appeared most suited to the diversity of studies included in the systematic review. We were required to modify the critical appraisal tool for the single-arm studies to ensure a comprehensive RoB assessment. Commonly used RoB tools such as the ROBINS-I or Newcastle–Ottawa Scale and the Joanna Briggs tools used here do not provide an easy means of quantifying their findings.^16^ We therefore created cut-points for the RoB assessments to assist the reader; however, we recognise that this approach has not been validated and that the findings should be regarded with caution. The result of the RoB assessments was that the majority of studies had either low or moderate RoB. However, there were no obvious trends for the impact of the quality of included studies on outcome. The sample sizes of the included studies ranged from five to 235 participants. RoB and sample size are critical elements in determining the impact of individual studies. Owing to study heterogeneity, we did not complete a meta-analysis or test for publication bias but it is possible that among smaller studies, those with positive results are more likely to be put forward or accepted for publication.

In conclusion, the addition of psychotherapy to ketamine treatment for psychiatric disorders is associated with largely positive outcomes and appears to offer promise. The range of diagnoses, psychotherapies and treatment protocols identified by this review suggests that definitive recommendations for integrating psychotherapy into ketamine protocols cannot yet be made. We recommend that larger-scale RCTs are undertaken using manualised psychotherapies and consistent, reproduceable ketamine protocols to advance knowledge in this area. We also recommend that future studies are oriented to evaluate whether the addition of psychotherapy to ketamine treatment enhances short-term benefits and delays relapse.

## Data Availability

All available data are contained within the submitted work.

## Supplementary material

For supplementary material accompanying this paper visit http://doi.org/10.1192/bjo.2023.53. click here to view supplementary material
