# Supplementary material for: Ketamine and psychotherapy for the treatment of psychiatric disorders: systematic review
Source: BJPsych Open. 2023 May 2;9(3):e79. doi: 10.1192/bjo.2023.53 (PMC10228275; doi:10.1192/bjo.2023.53)
Supplement: Supplementary file 1 [file bjosup.zip › S2056472423000534sup001.docx]

JBI Critical Appraisal Checklist for
randomized Controlled trials

Author: Dakwar Year: 2019 Score: 12/13 = 92.3%, low ROB

|  | Yes | No | Unclear | NA |
| --- | --- | --- | --- | --- |
| 1. Was true randomization used for assignment of participants to treatment groups? | □ | □ | □ | □ |
| 1. Was allocation to treatment groups concealed? | □ | □ | □ | □ |
| 1. Were treatment groups similar at the baseline? | □ | □ | □ | □ |
| 1. Were participants blind to treatment assignment? | □ | □ | □ | □ |
| 1. Were those delivering treatment blind to treatment assignment? | □ | □ | □ | □ |
| 1. Were outcomes assessors blind to treatment assignment? | □ | □ | □ | □ |
| 1. Were treatment groups treated identically other than the intervention of interest? | □ | □ | □ | □ |
| 1. Was follow up complete and if not, were differences between groups in terms of their follow up adequately described and analyzed? | □ | □ | □ | □ |
| 1. Were participants analyzed in the groups to which they were randomized? | □ | □ | □ | □ |
| 1. Were outcomes measured in the same way for treatment groups? | □ | □ | □ | □ |
| 1. Were outcomes measured in a reliable way? | □ | □ | □ | □ |
| 1. Was appropriate statistical analysis used? | □ | □ | □ | □ |
| 1. Was the trial design appropriate, and any deviations from the standard RCT design (individual randomization, parallel groups) accounted for in the conduct and analysis of the trial? | □ | □ | □ | □ |

JBI Critical Appraisal Checklist for
randomized Controlled trials

Author Dakwar Year 2020 Score: 12/13 = 92.3%, Low ROB

|  | Yes | No | Unclear | NA |
| --- | --- | --- | --- | --- |
| 1. Was true randomization used for assignment of participants to treatment groups? | □ | □ | □ | □ |
| 1. Was allocation to treatment groups concealed? | □ | □ | □ | □ |
| 1. Were treatment groups similar at the baseline? | □ | □ | □ | □ |
| 1. Were participants blind to treatment assignment? | □ | □ | □ | □ |
| 1. Were those delivering treatment blind to treatment assignment? | □ | □ | □ | □ |
| 1. Were outcomes assessors blind to treatment assignment? | □ | □ | □ | □ |
| 1. Were treatment groups treated identically other than the intervention of interest? | □ | □ | □ | □ |
| 1. Was follow up complete and if not, were differences between groups in terms of their follow up adequately described and analyzed? | □ | □ | □ | □ |
| 1. Were participants analyzed in the groups to which they were randomized? | □ | □ | □ | □ |
| 1. Were outcomes measured in the same way for treatment groups? | □ | □ | □ | □ |
| 1. Were outcomes measured in a reliable way? | □ | □ | □ | □ |
| 1. Was appropriate statistical analysis used? | □ | □ | □ | □ |
| 1. Was the trial design appropriate, and any deviations from the standard RCT design (individual randomization, parallel groups) accounted for in the conduct and analysis of the trial? Consensus yes | □ | □ | □ | □ |

JBI Critical Appraisal Checklist for
randomized Controlled trials

Author: Grabski Year 2022 Score: 12/13 = 92.3% Low ROB

|  | Yes | No | Unclear | NA |
| --- | --- | --- | --- | --- |
| 1. Was true randomization used for assignment of participants to treatment groups? | □ | □ | □ | □ |
| 2. Was allocation to treatment groups concealed? | □ | □ | □ | □ |
| 1. Were treatment groups similar at the baseline? Consensus no | □ | □ | □ | □ |
| 1. Were participants blind to treatment assignment? | □ | □ | □ | □ |
| 1. Were those delivering treatment blind to treatment assignment? | □ | □ | □ | □ |
| 1. Were outcomes assessors blind to treatment assignment? | □ | □ | □ | □ |
| 1. Were treatment groups treated identically other than the intervention of interest? | □ | □ | □ | □ |
| 1. Was follow up complete and if not, were differences between groups in terms of their follow up adequately described and analyzed? | □ | □ | □ | □ |
| 1. Were participants analyzed in the groups to which they were randomized? | □ | □ | □ | □ |
| 1. Were outcomes measured in the same way for treatment groups? | □ | □ | □ | □ |
| 1. Were outcomes measured in a reliable way? | □ | □ | □ | □ |
| 1. Was appropriate statistical analysis used? | □ | □ | □ | □ |
| 1. Was the trial design appropriate, and any deviations from the standard RCT design (individual randomization, parallel groups) accounted for in the conduct and analysis of the trial? | □ | □ | □ | □ |

JBI Critical Appraisal Checklist for
quasi-experimental studies

Author Krupitsky Year 1996 Rating:: 6/9 = 66.6%, Moderate ROB

|  | Yes | No | Unclear | Not applicable |
| --- | --- | --- | --- | --- |
| 1. Is it clear in the study what is the ‘cause’ and what is the ‘effect’ (i.e. there is no confusion about which variable comes first)? | □ | □ | □ | □ |
| 1. Were the participants included in any comparisons similar? | □ | □ | □ | □ |
| 1. Were the participants included in any comparisons receiving similar treatment/care, other than the exposure or intervention of interest? | □ | □ | □ | □ |
| 1. Was there a control group? | □ | □ | □ | □ |
| 1. Were there multiple measurements of the outcome both pre and post the intervention/exposure? | □ | □ | □ | □ |
| 1. Was follow up complete and if not, were differences between groups in terms of their follow up adequately described and analyzed? | □ | □ | □ | □ |
| 1. Were the outcomes of participants included in any comparisons measured in the same way? | □ | □ | □ | □ |
| 1. Were outcomes measured in a reliable way? | □ | □ | □ | □ |
| 1. Was appropriate statistical analysis used? | □ | □ | □ | □ |

JBI Critical Appraisal Checklist for
randomized Controlled trials

Author Krupistky Year 2007 Score: 10/13 = 76.9%, Low ROB

|  | Yes | No | Unclear | NA |
| --- | --- | --- | --- | --- |
| 1. Was true randomization used for assignment of participants to treatment groups? | □ | □ | □ | □ |
| 1. Was allocation to treatment groups concealed? | □ | □ | □ | □ |
| 1. Were treatment groups similar at the baseline? | □ | □ | □ | □ |
| 1. Were participants blind to treatment assignment? | □ | □ | □ | □ |
| 1. Were those delivering treatment blind to treatment assignment? | □ | □ | □ | □ |
| 1. Were outcomes assessors blind to treatment assignment? | □ | □ | □ | □ |
| 1. Were treatment groups treated identically other than the intervention of interest? | □ | □ | □ | □ |
| 1. Was follow up complete and if not, were differences between groups in terms of their follow up adequately described and analyzed? | □ | □ | □ | □ |
| 1. Were participants analyzed in the groups to which they were randomized? | □ | □ | □ | □ |
| 1. Were outcomes measured in the same way for treatment groups? | □ | □ | □ | □ |
| 1. Were outcomes measured in a reliable way? | □ | □ | □ | □ |
| 1. Was appropriate statistical analysis used? | □ | □ | □ | □ |
| 1. Was the trial design appropriate, and any deviations from the standard RCT design (individual randomization, parallel groups) accounted for in the conduct and analysis of the trial? | □ | □ | □ | □ |

JBI Critical Appraisal Checklist for
randomized Controlled trials

Author_ Krupistky Year 2002 Score: 12/13 = 92.3%, Low ROB

|  | Yes | No | Unclear | NA |
| --- | --- | --- | --- | --- |
| 1. Was true randomization used for assignment of participants to treatment groups? | □ | □ | □ | □ |
| 1. Was allocation to treatment groups concealed? | □ | □ | □ | □ |
| 1. Were treatment groups similar at the baseline? | □ | □ | □ | □ |
| 1. Were participants blind to treatment assignment? | □ | □ | □ | □ |
| 1. Were those delivering treatment blind to treatment assignment? | □ | □ | □ | □ |
| 1. Were outcomes assessors blind to treatment assignment? | □ | □ | □ | □ |
| 1. Were treatment groups treated identically other than the intervention of interest? | □ | □ | □ | □ |
| 1. Was follow up complete and if not, were differences between groups in terms of their follow up adequately described and analyzed? | □ | □ | □ | □ |
| 1. Were participants analyzed in the groups to which they were randomized? | □ | □ | □ | □ |
| 1. Were outcomes measured in the same way for treatment groups? | □ | □ | □ | □ |
| 1. Were outcomes measured in a reliable way? | □ | □ | □ | □ |
| 1. Was appropriate statistical analysis used? | □ | □ | □ | □ |
| 1. Was the trial design appropriate, and any deviations from the standard RCT design (individual randomization, parallel groups) accounted for in the conduct and analysis of the trial? Consensus yes | □ | □ | □ | □ |

JBI Critical Appraisal Checklist for
randomized Controlled trials

Author Pradhan Year 2017 Score: 9/13 = 69.2%, Moderate ROB

|  | Yes | No | Unclear | NA |
| --- | --- | --- | --- | --- |
| 1. Was true randomization used for assignment of participants to treatment groups? | □ | □ | □ | □ |
| 1. Was allocation to treatment groups concealed? | □ | □ | □ | □ |
| 1. Were treatment groups similar at the baseline? | □ | □ | □ | □ |
| 1. Were participants blind to treatment assignment? | □ | □ | □ | □ |
| 1. Were those delivering treatment blind to treatment assignment? | □ | □ | □ | □ |
| 1. Were outcomes assessors blind to treatment assignment? | □ | □ | □ | □ |
| 1. Were treatment groups treated identically other than the intervention of interest? | □ | □ | □ | □ |
| 1. Was follow up complete and if not, were differences between groups in terms of their follow up adequately described and analyzed? | □ | □ | □ | □ |
| 1. Were participants analyzed in the groups to which they were randomized? | □ | □ | □ | □ |
| 1. Were outcomes measured in the same way for treatment groups? | □ | □ | □ | □ |
| 1. Were outcomes measured in a reliable way? | □ | □ | □ | □ |
| 1. Was appropriate statistical analysis used? Consensus No | □ | □ | □ | □ |
| 1. Was the trial design appropriate, and any deviations from the standard RCT design (individual randomization, parallel groups) accounted for in the conduct and analysis of the trial? Consensus No | □ | □ | □ | □ |

JBI Critical Appraisal Checklist for
randomized Controlled trials

Author Pradhan Year 2018 Score: 12/13 = 92.3%, low ROB

|  | Yes | No | Unclear | NA |
| --- | --- | --- | --- | --- |
| 1. Was true randomization used for assignment of participants to treatment groups? | □ | □ | □ | □ |
| 1. Was allocation to treatment groups concealed? | □ | □ | □ | □ |
| 1. Were treatment groups similar at the baseline? | □ | □ | □ | □ |
| 1. Were participants blind to treatment assignment? | □ | □ | □ | □ |
| 1. Were those delivering treatment blind to treatment assignment? | □ | □ | □ | □ |
| 1. Were outcomes assessors blind to treatment assignment? | □ | □ | □ | □ |
| 1. Were treatment groups treated identically other than the intervention of interest? | □ | □ | □ | □ |
| 1. Was follow up complete and if not, were differences between groups in terms of their follow up adequately described and analyzed? | □ | □ | □ | □ |
| 1. Were participants analyzed in the groups to which they were randomized? | □ | □ | □ | □ |
| 1. Were outcomes measured in the same way for treatment groups? | □ | □ | □ | □ |
| 1. Were outcomes measured in a reliable way? | □ | □ | □ | □ |
| 1. Was appropriate statistical analysis used? | □ | □ | □ | □ |
| 1. Was the trial design appropriate, and any deviations from the standard RCT design (individual randomization, parallel groups) accounted for in the conduct and analysis of the trial? | □ | □ | □ | □ |

JBI Critical Appraisal Checklist for
randomized Controlled trials

Author Wilkinson Year 2021 Score: 9/13 = 69.2%, moderate ROB

|  | Yes | No | Unclear | NA |
| --- | --- | --- | --- | --- |
| 1. Was true randomization used for assignment of participants to treatment groups? | □ | □ | □ | □ |
| 1. Was allocation to treatment groups concealed? | □ | □ | □ | □ |
| 1. Were treatment groups similar at the baseline? | □ | □ | □ | □ |
| 1. Were participants blind to treatment assignment? | □ | □ | □ | □ |
| 1. Were those delivering treatment blind to treatment assignment? | □ | □ | □ | □ |
| 1. Were outcomes assessors blind to treatment assignment? | □ | □ | □ | □ |
| 1. Were treatment groups treated identically other than the intervention of interest? | □ | □ | □ | □ |
| 1. Was follow up complete and if not, were differences between groups in terms of their follow up adequately described and analyzed? | □ | □ | □ | □ |
| 1. Were participants analyzed in the groups to which they were randomized? | □ | □ | □ | □ |
| 1. Were outcomes measured in the same way for treatment groups? | □ | □ | □ | □ |
| 1. Were outcomes measured in a reliable way? | □ | □ | □ | □ |
| 1. Was appropriate statistical analysis used? | □ | □ | □ | □ |
| 1. Was the trial design appropriate, and any deviations from the standard RCT design (individual randomization, parallel groups) accounted for in the conduct and analysis of the trial? | □ | □ | □ | □ |

**Adapted Critical Joanna Briggs Appraisal Tools for Single Arm (pre-post) studies.**

Author Azhari Year 2021 Score: 6/10 = 60%, Moderate ROB

|  | Yes | No | Unclear | Not applicable |
| --- | --- | --- | --- | --- |
| Were the criteria for inclusion and exclusion in the study clearly defined? | □ | □ | □ | □ |
| Were the patient’s demographic and clinical information described in detail? | □ | □ | □ | □ |
| Were objective, standardised criteria used for measurement of the condition? | □ | □ | □ | □ |
| Was the intervention and setting described in detail? | □ | □ | □ | □ |
| Did all participants receive the same intervention protocol? | □ | □ | □ | □ |
| Were confounding factors identified? | □ | □ | □ | □ |
| Were strategies to account for confounding factors identified? | □ | □ | □ | □ |
| Were outcomes measured in a valid and reliable way? | □ | □ | □ | □ |
| Did all participants complete the intervention, and if not were missing data adequately described and analysed? | □ | □ | □ | □ |
| Was the statistical analysis appropriate? | □ | □ | □ | □ |

**Adapted Critical Joanna Briggs Appraisal Tools for Single Arm (pre-post) studies.**

Author Dames Year 2022 Score: 2/10 = 0%, High ROB

|  | Yes | No | Unclear | Not applicable |
| --- | --- | --- | --- | --- |
| Were the criteria for inclusion and exclusion in the study clearly defined? | □ | □ | □ | □ |
| Were the patient’s demographic and clinical information described in detail? | □ | □ | □ | □ |
| Were objective, standardised criteria used for measurement of the condition? | □ | □ | □ | □ |
| Was the intervention and setting described in detail? | □ | □ | □ | □ |
| Did all participants receive the same intervention protocol? | □ | □ | □ | □ |
| Were confounding factors identified? | □ | □ | □ | □ |
| Were strategies to account for confounding factors identified? | □ | □ | □ | □ |
| Were outcomes measured in a valid and reliable way? | □ | □ | □ | □ |
| Did all participants complete the intervention, and if not were missing data adequately described and analysed? | □ | □ | □ | □ |
| Was the statistical analysis appropriate? | □ | □ | □ | □ |

**Adapted Critical Joanna Briggs Appraisal Tools for Single Arm (pre-post) studies.**

Author Davis Year 2021 Score: 5/10 = 50%, moderate ROB

|  | Yes | No | Unclear | Not applicable |
| --- | --- | --- | --- | --- |
| Were the criteria for inclusion and exclusion in the study clearly defined? | □ | □ | □ | □ |
| Were the patient’s demographic and clinical information described in detail? | □ | □ | □ | □ |
| Were objective, standardised criteria used for measurement of the condition? | □ | □ | □ | □ |
| Was the intervention and setting described in detail? | □ | □ | □ | □ |
| Did all participants receive the same intervention protocol? | □ | □ | □ | □ |
| Were confounding factors identified? | □ | □ | □ | □ |
| Were strategies to account for confounding factors identified? | □ | □ | □ | □ |
| Were outcomes measured in a valid and reliable way? | □ | □ | □ | □ |
| Did all participants complete the intervention, and if not were missing data adequately described and analysed? | □ | □ | □ | □ |
| Was the statistical analysis appropriate? | □ | □ | □ | □ |

**Adapted Critical Joanna Briggs Appraisal Tools for Single Arm (pre-post) studies.**

Author Dore Year 2019 Score: 3/10 = 30%, high ROB

|  | Yes | No | Unclear | Not applicable |
| --- | --- | --- | --- | --- |
| Were the criteria for inclusion and exclusion in the study clearly defined? | □ | □ | □ | □ |
| Were the patient’s demographic and clinical information described in detail? | □ | □ | □ | □ |
| Were objective, standardised criteria used for measurement of the condition? | □ | □ | □ | □ |
| Was the intervention and setting described in detail? | □ | □ | □ | □ |
| Did all participants receive the same intervention protocol? | □ | □ | □ | □ |
| Were confounding factors identified? | □ | □ | □ | □ |
| Were strategies to account for confounding factors identified? | □ | □ | □ | □ |
| Were outcomes measured in a valid and reliable way? | □ | □ | □ | □ |
| Did all participants complete the intervention, and if not were missing data adequately described and analysed? | □ | □ | □ | □ |
| Was the statistical analysis appropriate? | □ | □ | □ | □ |

**Adapted Critical Joanna Briggs Appraisal Tools for Single Arm (pre-post) studies.**

Author Keizer Year 2020 Score: 3/10 = 50%, high ROB

|  | Yes | No | Unclear | Not applicable |
| --- | --- | --- | --- | --- |
| Were the criteria for inclusion and exclusion in the study clearly defined? | □ | □ | □ | □ |
| Were the patient’s demographic and clinical information described in detail? In supplement | □ | □ | □ | □ |
| Were objective, standardised criteria used for measurement of the condition? | □ | □ | □ | □ |
| Was the intervention and setting described in detail? | □ | □ | □ | □ |
| Did all participants receive the same intervention protocol? See supplement | □ | □ | □ | □ |
| Were confounding factors identified? | □ | □ | □ | □ |
| Were strategies to account for confounding factors identified? | □ | □ | □ | □ |
| Were outcomes measured in a valid and reliable way? | □ | □ | □ | □ |
| Did all participants complete the intervention, and if not were missing data adequately described and analysed? Data missing for patient 3 and 4 | □ | □ | □ | □ |
| Was the statistical analysis appropriate? | □ | □ | □ | □ |

**JBI Critical Appraisal Checklist for Case Series**

Author Robison Year 2022 Score: 5/10 = 50%, moderate ROB

|  | Yes | No | Unclear | Not applicable |
| --- | --- | --- | --- | --- |
| Were the criteria for inclusion and exclusion in the study clearly defined? | □ | □ | □ | □ |
| Were the patient’s demographic and clinical information described in detail? | □ | □ | □ | □ |
| Were objective, standardised criteria used for measurement of the condition? | □ | □ | □ | □ |
| Was the intervention and setting described in detail? | □ | □ | □ | □ |
| Did all participants receive the same intervention protocol? | □ | □ | □ | □ |
| Were confounding factors identified? | □ | □ | □ | □ |
| Were strategies to account for confounding factors identified? | □ | □ | □ | □ |
| Were outcomes measured in a valid and reliable way? | □ | □ | □ | □ |
| Did all participants complete the intervention, and if not were missing data adequately described and analysed? | □ | □ | □ | □ |
| Was the statistical analysis appropriate? | □ | □ | □ | □ |

**Adapted Critical Joanna Briggs Appraisal Tools for Single Arm (pre-post) studies.**

Author Rodriguez Year 2016 Score: 5/10 = 50%, moderate ROB

|  | Yes | No | Unclear | Not applicable |
| --- | --- | --- | --- | --- |
| Were the criteria for inclusion and exclusion in the study clearly defined? | □ | □ | □ | □ |
| Were the patient’s demographic and clinical information described in detail? | □ | □ | □ | □ |
| Were objective, standardised criteria used for measurement of the condition? | □ | □ | □ | □ |
| Was the intervention and setting described in detail?  Not much detail? | □ | □ | □ | □ |
| Did all participants receive the same intervention protocol? | □ | □ | □ | □ |
| Were confounding factors identified? | □ | □ | □ | □ |
| Were strategies to account for confounding factors identified? | □ | □ | □ | □ |
| Were outcomes measured in a valid and reliable way? | □ | □ | □ | □ |
| Did all participants complete the intervention, and if not were missing data adequately described and analysed? | □ | □ | □ | □ |
| Was the statistical analysis appropriate? | □ | □ | □ | □ |

**Adapted Critical Joanna Briggs Appraisal Tools for Single Arm (pre-post) studies.**

Author Shrioma Year 2020 Score: 7/10 = 70%, moderate ROB

|  | Yes | No | Unclear | Not applicable |
| --- | --- | --- | --- | --- |
| Were the criteria for inclusion and exclusion in the study clearly defined? | □ | □ | □ | □ |
| Were the patient’s demographic and clinical information described in detail? | □ | □ | □ | □ |
| Were objective, standardised criteria used for measurement of the condition? | □ | □ | □ | □ |
| Was the intervention and setting described in detail? | □ | □ | □ | □ |
| Did all participants receive the same intervention protocol? | □ | □ | □ | □ |
| Were confounding factors identified? | □ | □ | □ | □ |
| Were strategies to account for confounding factors identified? | □ | □ | □ | □ |
| Were outcomes measured in a valid and reliable way? | □ | □ | □ | □ |
| Did all participants complete the intervention, and if not were missing data adequately described and analysed? Intention to treat analysis | □ | □ | □ | □ |
| Was the statistical analysis appropriate? | □ | □ | □ | □ |

**Adapted Critical Joanna Briggs Appraisal Tools for Single Arm (pre-post) studies.**

Author Wilkinson Year 2017 Score: 8/10 = 80%, low ROB

|  | Yes | No | Unclear | Not applicable |
| --- | --- | --- | --- | --- |
| Were the criteria for inclusion and exclusion in the study clearly defined? | □ | □ | □ | □ |
| Were the patient’s demographic and clinical information described in detail? | □ | □ | □ | □ |
| Were objective, standardised criteria used for measurement of the condition? MINI? | □ | □ | □ | □ |
| Was the intervention and setting described in detail? | □ | □ | □ | □ |
| Did all participants receive the same intervention protocol? | □ | □ | □ | □ |
| Were confounding factors identified? Which ones? | □ | □ | □ | □ |
| Were strategies to account for confounding factors identified? | □ | □ | □ | □ |
| Were outcomes measured in a valid and reliable way? | □ | □ | □ | □ |
| Did all participants complete the intervention, and if not were missing data adequately described and analysed? | □ | □ | □ | □ |
| Was the statistical analysis appropriate? | □ | □ | □ | □ |

**Adapted Critical Joanna Briggs Appraisal Tools for Single Arm (pre-post) studies.**

Author Zdyb Year 2021 Score: 6/10 = 60%, moderate ROB.

|  | Yes | No | Unclear | Not applicable |
| --- | --- | --- | --- | --- |
| Were the criteria for inclusion and exclusion in the study clearly defined? Exclusion criteria? | □ | □ | □ | □ |
| Were the patient’s demographic and clinical information described in detail? | □ | □ | □ | □ |
| Were objective, standardised criteria used for measurement of the condition? | □ | □ | □ | □ |
| Was the intervention and setting described in detail? Separate publication | □ | □ | □ | □ |
| Did all participants receive the same intervention protocol? | □ | □ | □ | □ |
| Were confounding factors identified? | □ | □ | □ | □ |
| Were strategies to account for confounding factors identified? | □ | □ | □ | □ |
| Were outcomes measured in a valid and reliable way? | □ | □ | □ | □ |
| Did all participants complete the intervention, and if not were missing data adequately described and analysed? | □ | □ | □ | □ |
| Was the statistical analysis appropriate? | □ | □ | □ | □ |
