# Supplementary material for: Ketamine and psychotherapy for the treatment of psychiatric disorders: systematic review
Source: BJPsych Open. 2023 May 2;9(3):e79. doi: 10.1192/bjo.2023.53 (PMC10228275; doi:10.1192/bjo.2023.53)
Supplement: Supplementary file 1 [file bjosup.zip › S2056472423000534sup002.docx]

**Search strings for each databases**

*For PsycINFO, Embase, Medline:*

1. ketamine/

2. ketamine*.tw

3. esketamine*.tw

4. S-ketamine*.tw

5. 1 or 2 or 3 or 4

6. exp psychotherapy/

7. psychotherapy*.tw

8. counselling*.tw

9. talk therapy*.tw

10. 6 or 7 or 8 or 9

11. 5 and 10

12. Limit 11 to English language and human

*For SCOPUS and Cochrane Library:*

(ketamine OR esketamine OR S-ketamine)

(psychotherapy OR talk therapy OR counselling OR counselling)

*For Google Scholar:*

With all of the words: ketamine, S-ketamine

With at least one of the words:

1. Psychotherapy
2. Talk therapy
3. Counselling
